# Plasmodesmata-Mediated Cell-to-Cell Communication in the Shoot Apical Meristem: How Stem Cells Talk

**DOI:** 10.3390/plants6010012

**Published:** 2017-03-01

**Authors:** Munenori Kitagawa, David Jackson

**Affiliations:** Cold Spring Harbor Laboratory, 1 Bungtown Road, Cold Spring Harbor, NY 11724, USA; mkitagaw@cshl.edu

**Keywords:** cell-to-cell communication, plasmodesmata, *Arabidopsis thaliana*, mobile transcription factors, mobile small RNAs, receptor-like kinase/protein, callose

## Abstract

Positional information is crucial for the determination of plant cell fates, and it is established based on coordinated cell-to-cell communication, which in turn is essential for plant growth and development. Plants have evolved a unique communication pathway, with tiny channels called plasmodesmata (PD) spanning the cell wall. PD interconnect most cells in the plant and generate a cytoplasmic continuum, to mediate short- and long-distance trafficking of various molecules. Cell-to-cell communication through PD plays a role in transmitting positional signals, however, the regulatory mechanisms of PD-mediated trafficking are still largely unknown. The induction and maintenance of stem cells in the shoot apical meristem (SAM) depends on PD-mediated cell-to-cell communication, hence, it is an optimal model for dissecting the regulatory mechanisms of PD-mediated cell-to-cell communication and its function in specifying cell fates. In this review, we summarize recent knowledge of PD-mediated cell-to-cell communication in the SAM, and discuss mechanisms underlying molecular trafficking through PD and its role in plant development.

## 1. Introduction

**Figure 1:**
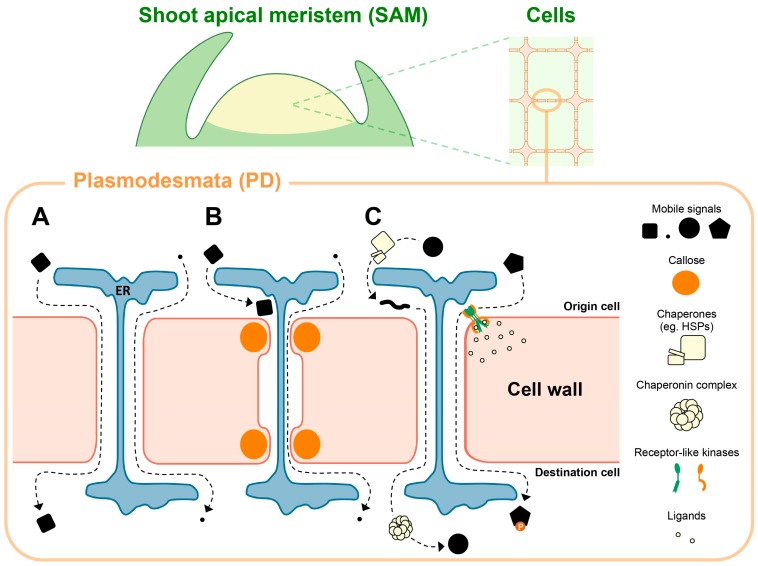
Plasmodesmata (PD)-mediated movement and its regulation. (**A**) PD are membrane-lined channels penetrating the cell wall, with desmotubules derived from endoplasmic reticulum (ER) (blue tubes). The cytosolic sleeve allows various micro- and macromolecules to move between cells (dashed lines). (**B**) PD permeability can be dynamically changed, for instance, by β-1,3-glucan (callose) deposition into PD orifice, leading to the reduction of PD pore size. (**C**) Specific macromolecular signals such as transcription factors (TFs) and small non-coding RNA (sRNAs) can be actively and selectively transported via PD. For example, some TFs may be unfolded by chaperones (e.g., heat shock proteins (HSPs)) to aid passage through PD, and then are refolded in the destination cells by the chaperonin complex. In addition, PD-located receptor-like kinases (RLKs) (and receptor-like proteins, RLPs) also regulate the molecular trafficking through PD. For example, they may respond to secreted ligands by phosphorylating non-cell autonomous proteins, to promote or restrict their trafficking, or to affect callose deposition.

For the development and growth of multicellular organisms, cell fates must be appropriately specified, and this is often achieved by using the positional information of each cell [1]. Such information is transmitted by coordinated cell-to-cell communication [2,3]. In plants, such communication sometimes occurs through transporters or receptor-ligand interactions, but plants have also developed a unique communication pathway through plasmodesmata (PD) [4]. PD are membrane-lined channels traversing the cell wall. A central strand of tightly compressed endoplasmic reticulum (ER), the so-called desmotubule, creates a region between the PM and ER membranes called the cytosolic sleeve, which provides continuity of cytoplasm between adjacent cells (Figure 1A) [5,6]. This continuity of cytoplasm is called the symplast, through which various molecules such as transcription factors (TFs), RNAs, and phytohormones can move [7,8,9,10,11]. Intercellular movement of molecules can be controlled depending on the structure and number of PD [12], and some macromolecules can be actively and selectively transported by interacting with a factor that can assist their movement [7,13]. Thus, PD-mediated cell-to-cell communication is dynamically regulated to transmit positional information between cells. However, when and how PD-mediated cell-to-cell communication is regulated in specifying cell fates is still largely unknown.

**Figure 2:**
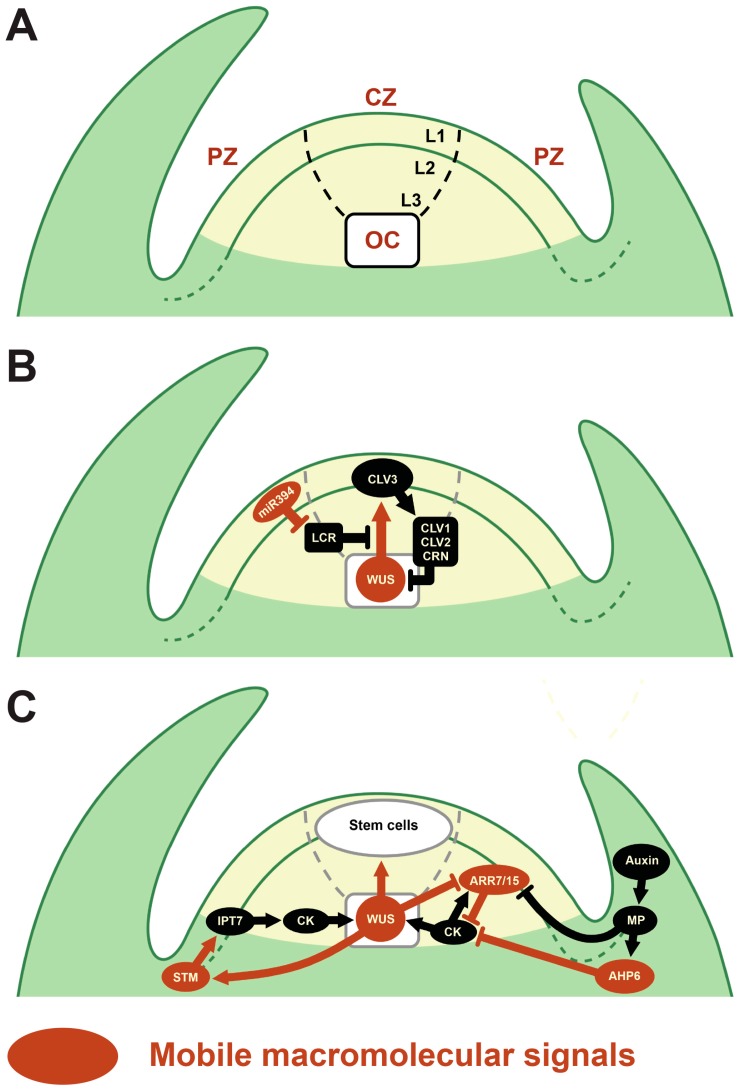
Shoot apical meristem (SAM) and mobile macromolecular signals. (**A**) In the SAM, stem cells are in the central zone (CZ) in three clonally distinct layers (L1–L3). At the base of the CZ, the organizing center (OC) induces and maintains the stem cells above. (**B**) The homeodomain (HD) TF WUSCHEL (WUS) is expressed in the OC and moves to the CZ to promote stem cell identity, in part by activating *CLAVATA3* (*CLV3*). *CLV3* encodes a small secreted peptide that is perceived by CLAVATA 1 (CLV1) and CLAVATA 2 (CLV2)/ CORYNE (CRN) to repress WUS expression and form a negative feedback loop to maintain the size of the stem cell pool. A microRNA (miRNA), miR394, is expressed in the L1 and moves to L2 and L3 to repress the expression of *LEAF CURLING RESPONSIVENESS* (*LCR*), promoting WUS function. (**C**) WUS positively regulates SHOOT MERISTEMLESS (STM) expression, which in turn promotes cytokinin (CK) synthesis by activating expression of ISOPENTENYLTRANSFERASE 7 (IPT7). WUS also directly represses type-A ARABIDOPSIS RESPONSE REGULATOR (ARR) 7 and ARR15, resulting in the establishment of a CK maximum in the OC, which in turn can activate WUS expression. ARABIDOPSIS HISTIDINE PHOSPHOTRANSFER PROTEIN 6 (AHP6) is expressed in organ primordia, and its intercellular movement forms a gradient centered on organ primordia and extending beyond their boundaries, which acts a CK-signaling inhibitory field, to enhance robustness of phyllotaxis. All cells in the SAM are connected by plasmodesmata (as drawn in Figure 1).

A connection between PD-mediated signaling and cell fates is in their role in initiation and maintenance of stem cells during plant development [14]. In plants, stem cells are situated within meristems that allow long term self-renewal potential [15]. The shoot apical meristems (SAMs) are one class of plant meristems, and give rise to all shoot tissues (leaves, stems, flowers, and germline). The stem cells within the SAMs are found in the central zone (CZ) composed of a variable number of clonally distinct cell layers; L1, L2, and L3 in most plants, including *Arabidopsis*. Stem cells in the L1 generate epidermal tissue and some underlying tissues, and those of L2 and L3 generate ground and vascular tissues (Figure 2A). Under the CZ is the organizing center (OC), which induces and maintains the stem cells above. When stem cells divide, their progeny are pushed laterally into the peripheral zone (PZ) where they rapidly divide to generate cells that build the lateral organs or the stem. Thus, plant cells are passively displaced by tissue growth driven by cell division, and their fates are determined by their eventual position, and therefore must be dependent on positional signals. Indeed, recent studies revealed that this positional information is established by PD-mediated cell-to-cell communication in the SAMs, and is crucial for cellular differentiation and stem cell maintenance [16,17]. In this review, we focus on recent progress regarding the control of mobile signals transported by PD and their function in the development of the SAMs.

## 2. Macromolecular Trafficking through PD in the SAM

### 2.1. Mobile TFs

Most cells in plant tissues cannot freely move, due to their rigid cell walls. In order to dynamically specify cell fates, positional determinants need to be transported to target cells and function correctly there. It is widely accepted that macromolecular signals such as TFs and small non-coding RNA (sRNAs) act as mobile signals that are actively and selectively transported to target cells through PD to specify their fates [18]. Around twenty years ago, the first mobile signal moving between cells via PD, the homeodomain (HD) TF KNOTTED1 (KN1), was discovered in maize. *KN1* is expressed in the L2 layers of the maize SAM, but KN1 protein moves into the L1 [19]. The KN1 homologs in *Arabidopsis*, SHOOT MERISTEMLESS (STM) and ARABIDOPSIS KNOTTED-LIKE (KNAT1)/BREVIPEDICELLUS (BP), also act as mobile proteins. These proteins can move from the L1 into the inner cell layers in the SAM when driven by an L1 specific promoter [20,21]. During normal development, KNAT1 moves from the cortex to epidermal cells in stems, and movement is required for the determination of plant architecture and epidermal differentiation [22]. Movement appears to require specific signals within these proteins, for example, the KN1 HD, composed of nuclear localization signal (NLS) and three helical domains, a defining feature of KN1-related homeobox (KNOX) family members [23], is necessary and sufficient for its PD-mediated transport [20].

KN1 and STM function in the initiation and maintenance of the SAM, and their ectopic expression results in the formation of ectopic meristems [24]. Thus, they are key TFs to specify stem cell fate during SAM development, but we do not know if their movement via PD is essential for their function. For *Arabidopsis* STM, in particular, there is no obvious difference between its mRNA and protein localization [21]. A recent study indicated that STM accumulates preferentially at the boundary of the SAM, depending on the mechanical stress generated by tissue folding, and contributes to organ separation during SAM development [25]. On the other hand, in situ hybridization has shown that *STM* mRNA is uniformly expressed in the SAM, except in developing leaf primordia [25,26,27,28]. Therefore, it is possible that STM protein is actively transported to the region between meristem and organs to define the boundary, and to constrain the meristematic region in the SAM.

In addition to KNOX proteins, the HD TF WUSCHEL (WUS) also moves between cells through PD to maintain the stem cell pool in the SAM [29,30] (Figure 2B). WUS is expressed in the OC and moves to the CZ cells to promoting their identity as stem cells, in part by activating *CLAVATA3* (*CLV3*) expression. *CLV3* encodes a small secreted peptide that is received by a receptor-like kinase (RLK) CLAVATA 1 (CLV1), and the receptor-like protein/membrane pseudokinase CLAVATA 2 (CLV2)/CORYNE (CRN) complex to repress WUS expression and form a negative feedback loop to maintain the size of the stem cell pool [31]. To test the significance of PD-mediated WUS movement, it was artificially restricted by fusing tandem repeats of green fluorescent protein (GFP) to increase its molecular weight, by fusing nuclear localization signals (NLS) to target it to the nucleus, or by artificially blocking the PD pores [29,30]. In each case, restriction of WUS transport resulted in stem cell depletion phenotypes similar to *wus* mutants [30], suggesting that WUS transport via PD is crucial for maintenance of the SAM stem cell pool.

The mechanism underlying WUS transport through PD is still largely unknown. However, it seems to be driven by active transport rather than simple diffusion. GFP-fused WUS (WUS-GFP) or the closely related WUS-RELATED HOMEOBOX 5 (WOX5-GFP) are readily transported from OC to CZ [30]. In contrast, GFP-fused WOX13, which is one of the most distant WOX gene family members, showed markedly reduced movement. Thus, WOX protein movement through PD can be actively regulated depending on its sequence. WUS contains three evolutionally conserved domains; HD, WUS-box, and ethylene-responsive element binding factor-associated amphiphilic repression (EAR)-like domain, but these are dispensable for WUS movement, whereas the non-conserved sequence between the HD and WUS-box negatively regulates WUS transport [30]. When this non-conserved sequence is replaced by a non-specific linker, WUS transport occurs more widely, from the OC throughout the shoot apex [30]. The non-conserved sequence is also involved in homodimer formation, suggesting that this process regulates PD-mediated WUS movement, although direct evidence is missing.

### 2.2. Other Mobile Proteins

In addition to the WUS/CLV3 pathway, WUS also plays a central role in cytokinin (CK)-dependent regulation of SAM activity [17] (Figure 2C). CK signaling promotes proliferation and inhibits the differentiation of stem cells in the SAM. Indeed, direct application of CK promotes WUS expression, leading to an enlarged SAM, whereas CK synthesis or sensing defective mutants have a smaller SAM [32,33]. In this CK-dependent regulation of SAM activity, WUS positively regulates STM expression, which in turn promotes CK synthesis by activating expression of the CK biosynthetic enzyme ISOPENTENYLTRANSFERASE 7 (IPT7) [33]. Additionally, WUS directly represses negative regulators of CK signaling, type-A ARABIDOPSIS RESPONSE REGULATOR (ARR) 7 and ARR15 [32]. This results in the establishment of a CK maximum in the OC, which in turn can activate WUS expression [34,35]. ARRs may be important for this WUS/CK feedback by finely controlling SAM activity through modulating CK signaling. Furthermore, their expression is repressed by auxin signaling, suggesting that ARRs could be at the center of crosstalk between CK and auxin signaling [36]. Recently, it was revealed that these ARRs might also act as mobile signals [37], since GFP-fused ARR7 artificially expressed in the L1 was transported to L2/L3 layers. At present, however, the role of ARRs movement in the SAM is unknown, but appears to be independent of CK signaling. Recently, it has also been reported that the CK signaling inhibitor ARABIDOPSIS HISTIDINE PHOSPHOTRANSFER PROTEIN 6 (AHP6) acts as a mobile signal to generate positional information during organ development in the SAM, to stabilize leaf initiation patterns, or phyllotaxy [38]. AHP6 is expressed specifically in organ primordia, where it is activated downstream of auxin by MONOPTEROS [38,39]. AHP6 protein then moves between cells to form a gradient centered on organ primordia and extending beyond their boundaries. This gradient of AHP6 acts as a CK-signaling inhibitory field to prevent co-initiation of organs and enhance robustness, contributing to normal phyllotaxis during shoot development. Thus, the cell-to-cell movement of AHPs provides positional information through generating CK signaling inhibitory fields, which suggests that movement of ARRs may also be important in meristem control [38].

Similar to AHP6 and ARRs, other non-TF proteins can also act as mobile signals. A recent study indicated that DWARF14 (D14), a receptor of strigolactone (SL) hormone in rice, is transported long distances through phloem and then moves into axillary meristems (AMs) to regulate the function of SLs in the development of AMs [40]. *D14* mRNA accumulates in vascular bundles and leaf primordia, whereas natively expressed GFP-fused D14 protein (D14-GFP) can be found in AMs, suggesting that it is mobile. To test this idea, intercellular movement of D14 was artificially inhibited by increasing its molecular weight in a triple GFP fusion (D14-3xGFP). This immobile version of D14 could not rescue the tiller growth derepression phenotype of *d14* mutants. Thus, the D14 receptor acts as a mobile protein to promote the function of SLs in axillary shoot development. This finding provides a novel concept that hormone receptors, in addition to hormones, can act as mobile signals, extending the importance of PD in plant hormone signaling.

### 2.3. Mobile Small Non-Coding RNAs

In addition to proteins, small non-coding RNAs (sRNAs) also act as mobile signals. Various non-cell autonomous sRNAs have been identified, and they provide positional information for regulating leaf polarity, root vascular patterning, meristem formation, shoot meristem maintenance, and female gametogenesis (reviewed in [8]). MicroRNAs (miRNAs) are an especially well-studied class of sRNAs that suppress the expression of target genes by mRNA cleavage, translational inhibition, or transcriptional silencing [41,42,43]. A recent study indicated that miR394 acts as a mobile signal in stem cell maintenance in the SAM [44] (Figure 2B). *MIR394B* was identified through an ethyl methanesulfonate (EMS) mutant screen for enhancers of a weak allele of *ARGONAUTE10* (*AGO10*). AGO10 plays important roles in maintenance of the SAM, by preventing the accumulation of miR165 and 166 to positively regulate mRNA levels of their target homeodomain-leucine zipper protein (HD-ZIP) III genes [45,46], and also by positively regulating WUS activity [47]. In the SAM, miR394 is expressed in the L1 and moves to L2 and L3 to repress the expression of its target gene *LEAF CURLING RESPONSIVENESS* (*LCR*) [44]. This repression of *LCR* by miR394 is crucial for stem cell maintenance, since the SAM terminates prematurely in miR394-deficient mutants or in plants expressing a miR394-resistant form of LCR. The precise function of LCR is unknown, but it encodes a F-box protein, suggesting it is involved in protein degradation by the 26S proteasome [48]; however, its target is unlikely to be WUS, because LCR up-regulation did not affect WUS levels. However, LCR might target yet-unknown proteins that modify WUS function or transport. Thus, in addition to WUS trafficking from OC to CZ, the PD-mediated movement of miR394 from L1 to inner cell layers to repress *LCR* expression is crucial for the maintenance of stem cells in the SAM.

## 3. Regulatory Mechanisms of PD Movement

### 3.1. Chaperonin-Mediated TF Movement via PD

As described above, PD-mediated movement of macromolecular signals is important for development, but the mechanisms are still largely unknown. KN1 has been a model protein in studying the mechanisms underlying PD-mediated movement of TFs [20]. Previously, microinjection of structurally constrained KN1 protein revealed that movement was blocked by fixing its tertiary protein structure, suggesting that proteins are at least partially unfolded during movement between cells [49]. This model was further supported by the isolation of a mutant in intercellular movement of KN1 [50]. The mutant gene encoded CHAPERONIN CONTAINING T-COMPLEX POLYPEPTIDE 1 (CCT) 8, a subunit of a type II chaperonin. CCT8 can physically associate with KN1, STM, and TRANSPARENT TESTA GLABROUS1 (TTG1), a tryptophan-aspartic acid 40 (WD40)-repeat protein whose movement is involved in trichome spacing [51], and these interactions are required for their movement between cells through PD. Furthermore, *cct8* mutants enhance the stem cell depletion phenotype in a weak *stm* allele, suggesting that CCT8 mediated STM trafficking is required for stem cell maintenance in the SAM [50]. *Arabidopsis* possesses eight chaperonin subunits (CCT1-8), and at least one other, CCT7, physically associates with KN1, and both CCT1 and CCT5 are required for KN1 trafficking, suggesting that the whole chaperonin complex folds mobile proteins to facilitate their movement via PD. Moreover, it seems to be specifically required in “destination” cells for KN1 trafficking, suggesting that the chaperonin complex functions in the refolding of KN1 after PD transport.

CCT8 function is unlikely to be universally required for the movement of TFs through PD, however. For example, *cct8* mutants do not impact the movement of SHORT ROOT (SHR), a mobile TF critical to root patterning [50]. SHR is expressed in stele cells in the root and moves to endodermis, quiescent center, and the cortical endodermal initial cells to specify distinct endodermis and cortex cell fates [52,53,54]. SHR movement through PD is mediated by an endosome-associated protein, SHORT-ROOT INTERACTING EMBRYONIC LETHAL (SIEL) [55], and intact microtubules and the correct localization of SHR and SIEL at endosomes are required for PD-mediated SHR movement [56,57]. These findings suggest that microtubules assist in the movement of certain TFs to PD, and endosomes can be a platform for SIEL-dependent TF movement. Furthermore, while CCT8 associates with KN1, STM, and TTG [50], SIEL associates with SHR and three different mobile TFs, CAPRICE (CPC), TARGET OF MONOPTEROS 7 (TMO7), and AGAMOUS-LIKE 21 (AGL21) [55], although it is still unclear if SIEL promotes the movement of these additional proteins. Thus, there appears to be multiple different mechanisms underlying PD-mediated movement of TFs, which might be dependent on tissue type, developmental stage, or the structure of mobile proteins.

### 3.2. Regulation of PD Permeability and Frequency

While some proteins and sRNAs actively and selectively move through PD, as described above, it is also widely accepted that some small and large molecules can move non-specifically by simple diffusion [58,59]. This diffusion mediated movement is regulated by PD frequency and structure [12]. It is well-established that the deposition or degradation of β-1,3-glucan (callose) polymer at PD results in the modification of their aperture, leading to the alteration of PD permeability [60,61]. When callose accumulates at PD, the rate of non-specific trafficking is decreased. In contrast, when pore size is increased by the degradation of PD-associated callose, trafficking is increased. This callose-dependent regulation of PD permeability is dynamically controlled by callose synthases (GLUCAN SYNTHASE-LIKE (GSLs)/CALLOSE SYNTHASES (CALS)) [62] and callose degradation enzymes (β-1,3-GLUCANASES) [60,63,64], which modulate PD-mediated cell-to-cell communication during normal development and environmental responses. PD permeability can also be regulated by callose-independent pathways. For example, PD-localized proteins such as the Ca^2+^-binding protein calreticulin [65], cytoskeletal proteins [56,66,67], as well as reactive oxygen species (ROS) signaling (which can induce callose, or can function independently [68]) have been identified as factors that regulate PD permeability. Thus, the mechanisms underlying regulation of non-specific diffusion of molecules through PD are diverse, and alteration of PD permeability and frequency can establish specific groups of cells symplastically isolated from surrounding cells, in so-called symplastic domains [12]. These domains may restrict cell fate signals within specific groups of cells, leading to the establishment of tissue- or region-specific developmental programs [12].

The regulation of PD permeability is especially dynamic in the SAM [12], where the establishment of symplastic domains may be crucial for the maintenance of stem cell fate and dynamic changes of fate dependent on cell positions. To analyze such symplastic domains, fluorescent dyes can be introduced into a single cell, followed by observation of their intercellular diffusion though PD [69]. For example, microinjection of Lucifer yellow (LYCH, molecular weight 457 Da) into cells of the vegetative SAM of birch revealed that PD permeability between CZ and PZ is restricted [70,71]. Additionally, the movement of 8-Hydroxypyrene-1,3,6-trisulfonic acid (HPTS, molecular weight 524 Da) loaded from cut petioles of *Arabidopsis* leaves indicated that PD permeability at the interface between the phloem and the SAM is restricted in seedling stages, but increases prior to flowering [72]. At all stages, however, the region around the organizing center is symplastically isolated. Thus, PD permeability in the SAM is highly regulated depending on region and developmental stage, and this may establish symplastic domains to facilitate stem cell maintenance or differentiation.

PD permeability in perennial plants is also dynamically modified depending on the seasons. In the SAM of birch, for instance, short day conditions trigger PD closure through callose deposition, leading to SAM dormancy [70,71]. PD closure is maintained during dormancy, but the PD-associated callose is later degraded by β-1,3-GLUCANASES during chilling-induced dormancy release, leading to the opening of PD and the re-activation of the meristem [70,71]. This regulation of PD permeability may be important in controlling transduction of signals involved in dormancy of perennial plants. For example, FLOWERING LOCUS T (FT) is a mobile protein which is transported from leaves to the shoot apex to promote flowering, and acts as a negative regulator of dormancy [73]. Short day induced PD closure likely inhibits transport of FT and other signals into the meristem, to facilitate dormancy.

In addition to the alteration of PD permeability, movement may also be affected by the modification of PD frequency in the SAM. PD can be classified into primary PD and secondary PD, depending on the time of their formation; primary PD are formed by the trapping of ER by the newly forming cell plate during cell division, whereas secondary PD are generated de novo in post-cytokinetic walls as cells expand and differentiate [74]. The insertion of secondary PD and the removal of existing PD allow dynamic alteration of PD frequency during plant development [12,72]. For example, PD frequency is increased during the floral transition in all layers of the *Sinapis alba* SAM by the insertion of secondary PD, but returns to normal after the transition [75,76]. The molecular mechanisms and role of such dynamic modification of PD frequency are unknown. Recently, however, it has been reported that a choline transporter is involved in the insertion of secondary PD during *Arabidopsis* development [77]. Mutants in *CHOLINE TRANSPORTER-LIKE 1* (*CHER1*), which encodes a choline transporter, have altered the localization of a plant virus movement protein at PD. The *cher1* mutant, or down-regulation of *CHER1* expression, reduces secondary PD in the SAM and leaves. The frequency of complex, highly branched PD generated during cellular differentiation [78] are also reduced in *cher1* mutants. Thus, *CHER1* is crucial for the formation and maturation of secondary PD, perhaps by altering membrane composition important for PD formation or the targeting of proteins to PD, as suggested by other studies [79].

## 4. Regulation of PD-Mediated Movement by Receptors

Recently, some receptor-like kinases (RLKs) and receptor-like proteins (RLPs) have been localized to PD, where they modulate PD-mediated movement [80]. Many of these were identified by proteomic analysis of PD enriched plasma membrane (PM) fractions in *Arabidopsis* [81]. RLKs were also identified in a rice cell-wall proteome, and six of them were found to associate with PD when tagged using fluorescent proteins [82]. The functions of these RLKs and RLPs at PD are best understood in defense responses. For example, a RLP LYSIN MOTIF DOMAIN-CONTAINING GPI-ANCHORED PROTEIN (LYM2) and a RLK FLAGELLIN SENSING 2 (FLS2) facilitate PD closure by perception of microbial elicitors, chitin and flg22, respectively [83]. Another PD localized RLP, PDLP5, is upregulated by the defense hormone salicylic acid (SA), and promotes callose deposition in PD to reduce their permeability [84]. This controlled down-regulation of PD permeability by RLKs and RLPs may promote resistance against pathogens by modulating the intercellular transduction of systemic acquired resistance (SAR) signals [83,85,86]. Thus, the roles of RLKs and RLPs at PD during plant defense are well established, but their role in development and specification of cell fates is less clear [87].

CLAVATA 1 (CLV1) is one of the RLKs that may be involved in the developmental regulation of PD-mediated movement. In the SAM, CLV1 binds CLV3 peptide to repress WUS expression [31]. In contrast, in the root apical meristem, CLV1 perceives a peptide encoded by a CLV3 homolog CLAVATA3/EMBRYO SURROUNDING REGION40 (CLE40) to promote the differentiation of columella cells, and is associated with a second RLK, ARABIDOPSIS CRINKLY 4 (ACR4) at PD [88,89]. It is unclear whether this localization is required for cell fate determination, but one possibility is that it may restrict the trafficking of WUSCHEL-RELATED HOMEOBOX 5 (WOX5), and its role in maintaining stemness of cells [90]. In the SAM, ACR4 is expressed in the L1 [91], but it has no obvious loss of function phenotype [89]. However, it is possible that CLV1 associates with other RLPs or RLKs at the peripheral region of the OC to restrict WUS trafficking and regulate stem cell fate.

STRUBBELIG (SUB)/SCRAMBLED (SCM) is also a PD-localized RLK that controls morphogenesis of various organs such as flowers, stems, and roots [92,93,94,95,96,97]. SUB/SCM physically associates with the membrane-anchored C2-domain protein QUIRKY (QKY) at PD to non-cell autonomously regulate the shape of flowers and leaves, and integument development [98]. The SUB/QKY complex is also required for the regulation of cell growth and division plane [93,98], since shoot meristems of *sub* or *qky* mutants have altered cell shape, and L2 cells occasionally showed abnormal periclinal divisions. How the SUB/QKY complex non-cell autonomously regulates morphogenesis remains to be seen, but it has been proposed that it may mediate the PD trafficking of unknown mobile signals that function in cell fate determination [98]. In *sub* or *qky* mutants, the inter-cell layer diffusion of GFP through PD is not changed [98], suggesting that the SUB/QKY complex mediates active transport of specific mobile signals rather than general PD permeability. Since root hair patterning is defective in *sub* mutants [18,99], mobile TFs involved in the specification of root hair cells, such as CPC or GL3, are possible candidates, however, their movement was not altered in *sub* mutants [99]. Thus, further study is required to understand SUB/QKY-dependent signaling between cells. Important insights come from a QKY homolog, FT-INTERACTING PROTEIN 1 (FTIP1), which is localized to PD and functions in FT export from companion cells to sieve elements in the phloem. In the *ftip1* mutants, FT transport to shoot apex through phloem is compromised [100], leading to late flowering phenotypes even under long day floral induction conditions. This provides a further evidence that QKY is mechanistically involved in signal transport through PD.

Another class of PD-localized receptors is the PD LOCATED PROTEIN (PDLP) family, PD-located RLPs that contain a cytoplasmic domain, a single transmembrane domain, and two extracellular Domains of Unknown Function 26 (DUF26) [101]. As described above, one of them, PDLP5 facilitates callose deposition at PD to regulate PD permeability by SA signaling during plant-pathogen interactions [84]. Additionally, constitutive overexpression of PDLP1 induces callose deposition at PD, reducing permeability, whereas PD permeability is increased in knockout lines of several redundant PDLP clade members [101]. Thus, PDLPs act as a PD regulator, by promoting callose deposition at PD to change permeability. Among the eight PDLPs in *Arabidopsis*, PDLP2 and 3, in particular, have interesting expression patterns in the SAM [102]. PDLP3 is expressed in the L1 of the SAM and emerging floral primordia, and is higher in the PZ than in the CZ. On the other hand, PDLP2 is uniformly expressed within the inflorescence meristem, but is weaker in a boundary zone between floral primordia and the meristem. Double knock-out lines of PDLP2 and PDLP3 do not show detectable abnormalities in growth and development [102], suggesting that there is functional redundancy with other PDLPs. Hence, it is still unknown whether and how PDLPs act in non-cell autonomously specifying cell fates in the SAM, however, it is interesting that the expression pattern of PDLP3 resembles a symplastic domain defined by dye loading [102]. Moreover, the boundary region between meristem and lateral organs, where PDLP2 expression is reduced, corresponds to a region affected by mechanical stress generated from tissue folding, where boundary gene expression and auxin depletion are induced [25]. Therefore, PDLPs might regulate PD permeability in response to endogenous and exogenous signals to generate symplastic domains within the SAM.

## 5. Conclusions

Positional information guiding cell fate within the dynamic tissues of the SAM is established by the coordinated cell-to-cell communication. PD form one of the pathways mediating such communication between cells. PD-mediated movement of specific proteins and sRNAs is finely regulated by interactors such as the chaperonin complex, PD-located RLKs, and RLPs, and is crucial for the maintenance of the stem cell pool in the SAM. PD permeability and frequency are also dynamically modulated during SAM development, establishing symplastic domains that may control movement of stem cell or differentiation signals. There are exciting questions in PD-mediated signaling during plant development: How are macromolecules actively and selectively transported via PD? How are PD number and structure dynamically modified? How do PD-located RLKs and RLPs regulate PD-mediated signaling? The answers to these questions will be essential to understand the mechanism of development, morphogenesis, and growth in multicellular organisms that maintain symplastic communication.
